# A Newly Discovered *Obolenskvirus* Phage with Sustained Lytic Activity Against Multidrug-Resistant *Acinetobacter baumannii*

**DOI:** 10.3390/antibiotics14100961

**Published:** 2025-09-24

**Authors:** Eduardo Vera-Jauregui, María Guadalupe Avila-Novoa, Berenice González-Torres, Pedro Javier Guerrero-Medina, Cristobal Chaidez, Irvin González-López, Jean Pierre González-Gómez, Melesio Gutiérrez-Lomelí

**Affiliations:** 1 Centro de Investigación en Biotecnología Microbiana y Alimentaria, Departamento de Ciencias Básicas, División de Desarrollo Biotecnológico, Centro Universitario de la Ciénega, Universidad de Guadalajara, Av. Universidad 1115, Col. Lindavista, Ocotlan 47820, Mexico; eduardo.vjauregui@alumnos.udg.mx (E.V.-J.); avila.novoa@cuci.udg.mx (M.G.A.-N.); pjgm@cuci.udg.mx (P.J.G.-M.); 2 Laboratorio Nacional para la Investigación en Inocuidad Alimentaria (LANIIA), Centro de Investigación en Alimentación y Desarrollo (CIAD), Carretera a Eldorado Km 5.5, Campo El Diez, Culiacan 80110, Mexico; berenice.gonzalez@ciad.mx (B.G.-T.); chaqui@ciad.mx (C.C.); irvin.gonzalez@ciad.mx (I.G.-L.)

**Keywords:** phage therapy, multidrug resistance, *Acinetobacter baumannii*, *Obolenskvirus*

## Abstract

**Background**: *Acinetobacter baumannii* is a highly concerning pathogen in hospital settings, responsible for severe infections such as ventilator-associated pneumonia, urinary tract infections, and meningitis. Its remarkable genetic plasticity facilitates the rapid acquisition of antibiotic resistance, significantly complicating treatment and increasing mortality rates. As multidrug-resistant (MDR) infections continue to rise, phage therapy emerges as a viable alternative. **Methods**: This study reports the isolation and characterization of Acinetobacter phage vB_AbaM_A72 from stagnant water in Jalisco, Mexico. **Results**: Transmission electron microscopy revealed a myovirus-like morphology with an icosahedral head (91.32 ± 0.12 nm) and a contractile tail (123.77 ± 0.19 nm). The phage exhibited high environmental resilience, tolerating temperatures up to 60 °C and pH ranging from 5 to 11. Notably, A72 demonstrated a narrow host range but effectively inhibited the growth of an MDR *A. baumannii* strain for at least 12 h across different multiplicities of infection. Whole-genome sequencing confirmed the absence of virulence, antibiotic resistance, or lysogeny-associated genes. Comparative genomic analysis identified A72 as the first member of a newly described *Obolenskvirus* species, sharing only 76.4% similarity with its closest relatives. **Conclusions**: These findings underscore the importance of fully characterizing novel bacteriophages to expand therapeutic libraries and reinforce the feasibility of phage therapy as a promising approach against MDR *A. baumannii* infections.

## 1. Introduction

The world is facing a major threat: antibiotic resistance, a problem that is causing millions of deaths every year [1]. According to the criteria established by the Pan American Health Organization (PAHO), a microorganism is classified as multidrug-resistant (MDR) when the bacterial isolate shows in vitro resistance to at least one antimicrobial from three or more different classes of antibiotics [2]. Infections caused by these strains often result in inadequate or delayed treatment, which correlates with increased morbidity and mortality [3]. It is estimated that, by 2050, infections caused by these pathogens will exceed 10 million deaths, warning of a return to the “pre-antibiotic era”. This alert has led organizations such as the Centers for Disease Control (CDC) and the World Health Organization (WHO) to declare antibiotic resistance as a threat to global health and encouraged researchers to discover new alternatives for treating them [4,5].

The Infectious Diseases Society of America (IDSA) has highlighted a group of antimicrobial-resistant bacteria called ESKAPE pathogens (*Enterococcus faecium*, *Staphylococcus aureus*, *Klebsiella pneumoniae*, *Acinetobacter baumannii*, *Pseudomonas aeruginosa*, and *Enterobacter* spp.). This group has been noted for its ability to evade the biocidal activity of antibiotics through different mechanisms and represents new paradigms in pathogenesis, transmission, and resistance [6,7,8,9].

Particularly, *A. baumannii* is a non-fermenting Gram-negative coccobacillus belonging to the *Moraxellaceae* family. It has been found widely distributed in hospital and non-hospital environments such as soil, water, animals, and wastewater, among others [10]. This bacterium is an opportunistic pathogen responsible for nosocomial infections such as ventilator-associated pneumonia, urinary tract infections, meningitis, wound infections, and bacteremia, to name a few [11]. This pathogen has a high level of genetic plasticity that allows it to adopt different antimicrobial resistance phenotypes. One of these is the production of carbapenemases, especially OXA-51, OXA-23, and metallo-β-lactamases, which make treating infections by MDR strains even more difficult [12,13].

A very promising option for treating infections caused by multi-resistant strains of *A. baumannii* is the use of bacteriophages, a type of virus that exclusively infects bacteria and has even been used for a long time [4,14,15]. Phage therapy involves the direct administration of virulent phages to a patient to harness their lytic potential and eliminate the infection-causing pathogen [16,17]. This type of therapy has demonstrated both safety and efficacy in the treatment of human infections caused by *Escherichia coli*, *S. aureus*, *K. pneumoniae*, *E. faecium*, and even *A. baumannii* [18,19,20,21,22]. However, before starting to use it, it is necessary to characterize the isolated phage in order to provide sufficient evidence of their suitability for phage therapy. This phage must possess certain desirable characteristics to be used in phage therapy: (i) phage virulence (i.e., the ability to completely lyse a bacterial culture), (ii) a strictly lytic life cycle, (iii) transduction potential (i.e., the ability to transfer its genetic material to the host cell), (iv) absence of putatively harmful genes (toxins, antibiotic resistance, lysogeny, virulence, etc.), and (v) a broad host range within the same bacterial species. In addition, it is desired (but not essential) that it have an immunomodulatory effect, or the fitness cost of phage resistance be high, reducing the virulence of the bacteria or resensitizing it to antibiotics [23]. One of the crucial points for phage therapy is the availability of libraries with different, well-characterized phages and a diverse host range to cover the vast world of strain variety [24,25]. In this work, we seek to phenotypically and genomically characterize Acinetobacter phage vB_AbaM_A72, a unique phage belonging to the *Obolenskvirus* genus isolated from stagnant water that infects MDR *A. baumannii* strains.

## 2. Results

### 2.1. Isolation and General Features of Phages

**Figure 1:**
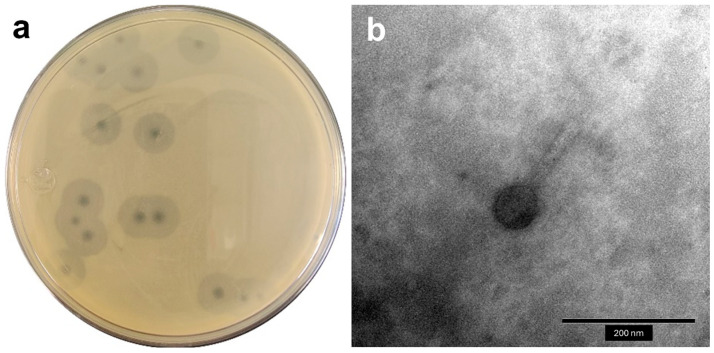
Macroscopic and microscopic morphology of the Acinetobacter phage vB_AbaM_A72. (**a**) Phage plaques on *A. baumannii* bacterial lawn. (**b**) TEM micrograph of the isolated phage.

The Acinetobacter phage vB_AbaM_A72 (phage A72 for short), named according to the new ICTV guidelines [26], was isolated from ponds using the enrichment method with MDR *A. baumannii* clinical strain A72 obtained from an endotracheal aspirate. Following purification, phage A72 produced clear plaques measuring 2–3 mm in diameter, surrounded by large turbid halos on double-layer agar bacterial lawn, as shown in Figure 1a). On the other hand, the morphology of the viral particles under TEM (Figure 1b) showed a myovirus morphology, with an icosahedral capsid with a diameter of 91.32 ± 0.12 nm and a contractile tail of 123.77 ± 0.19 nm (additional data are given in Figure S1).

### 2.2. Host Range Analysis and Efficacy of Plating (EOP)

In the host range analysis, the isolated phage demonstrated complete lysis (clear lysis zones) in only two of nineteen *A. baumannii* strains (10.5%), as shown in Table 1. What can be observed is that strains A19, A72, and A75 have the same antibiotic resistance profile, as shown in Table S1. To further evaluate the infectivity of phage A72, efficiency of plating (EOP) analysis was performed, and the EOP value was calculated. The phage exhibited high infectivity against A19 and A72 strains, based on the EOP results. Other strains belonging to different genera were tested, such as *S. aureus* ATCC 6538, *S. aureus* ATCC 25923, *E. coli* ATCC 11303, *S. enterica* ATCC 10708, *P. aeruginosa* ATCC 15,442, and *L. monocytogenes* ATCC 19111; however, A72 had no lytic activity against them.

### 2.3. Physiological Characterization

**Figure 2:**
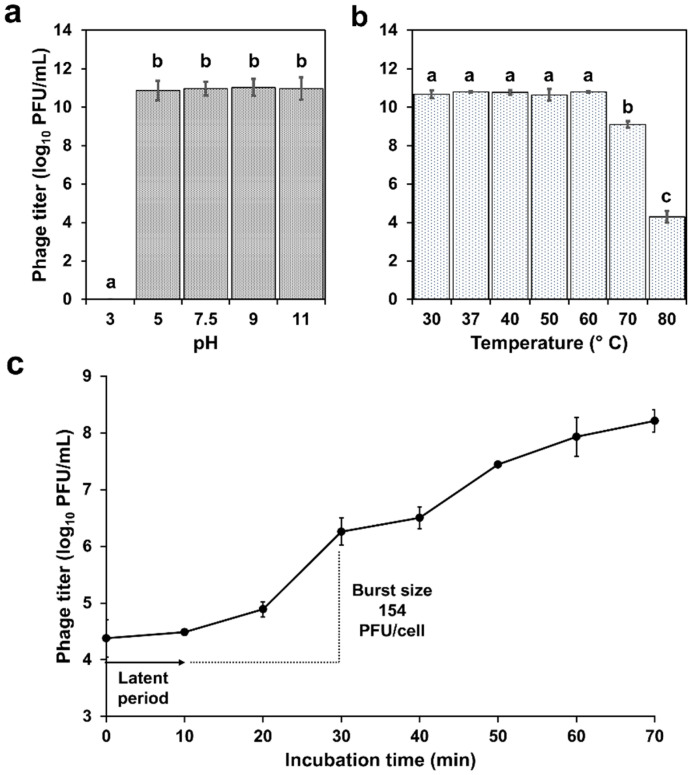
Physiological characterization and replication dynamics of Acinetobacter phage vB_AbaM_A72. (**a**) Stability of the phage at various pH values after 1 h of incubation. (**b**) Thermal stability of the phage after 1 h at different temperatures. (**c**) One-step growth curve showing the latent period and burst size of the phage. Values represent the means of three independent experiments ± standard deviation. Different letters in (**a**,**b**) indicate significant differences between treatments (*p* < 0.05).

The investigation of phage resistance at various pH revealed strong stability from pH 5 to 11, with total phage inactivation at pH 3 (Figure 2a).

On the other hand, the assessment of phage against different temperatures demonstrated robust stability between 30 and 60 °C, followed by a significant decline of approximately 100-fold at 70 °C. After exposure at 80 °C, the phage titer showed a reduction of 6 log_10_ PFU/mL (Figure 2b). In the one-step growth curve (Figure 2c), phage A72 showed a 12 min latent period and a burst size of approximately 154 viral particles per infected cell.

### 2.4. Bacteriolytic Activity

**Figure 3:**
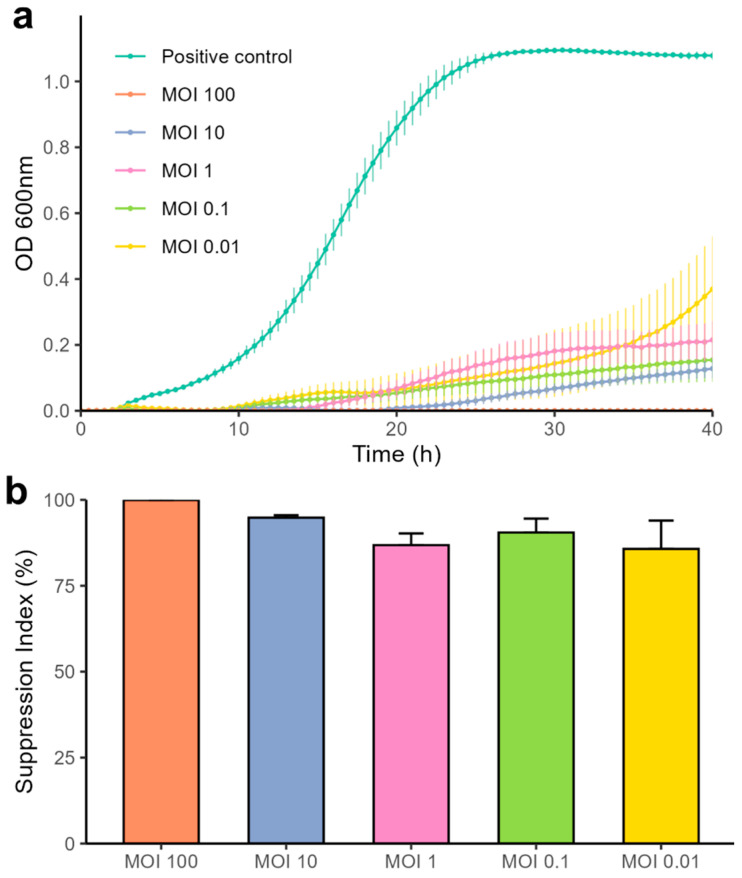
Bacteriolytic activity of Acinetobacter phage vB_AbaM_A72. (**a**) Bacterial growth of *A. baumannii* A72 exposed to different MOIs of phage A72. (**b**) Suppression index based on the area under the curve of the treatments against the positive control at 40 h of exposure. Values are the means of three tests ± SD.

The bacteriolytic activity of the isolated phage was evaluated by infecting strain A72 with varying multiplicities of infection, while bacterial growth was monitored via optical density measurements and compared against untreated controls. As shown in Figure 3, all tested MOIs significantly inhibited bacterial growth within the 12 h of exposure. The strongest suppressive effects were observed at MOI 100, which maintained complete bacterial suppression for the full 40 h incubation, and MOI 10, which prevented detectable growth for up to 20 h. Notably, all tested MOIs, including MOI 0.01, exhibited a suppression index above 80%, demonstrating robust lytic activity across infection doses. MOI 100 sustained 100% suppression throughout the experiment.

### 2.5. Genome Sequencing and Bioinformatic Analysis

Genome sequencing of Acinetobacter phage vB_AbaM_A72 showed a genome length of 44,172 base pairs with a G+C content of 37.7%. The genome included 92 coding sequences (CDS) and no tRNAs. The phage has a strictly lytic/virulent life cycle with no genes associated with lysogeny. Furthermore, no antibiotic resistance or virulence genes were detected.

**Figure 4:**
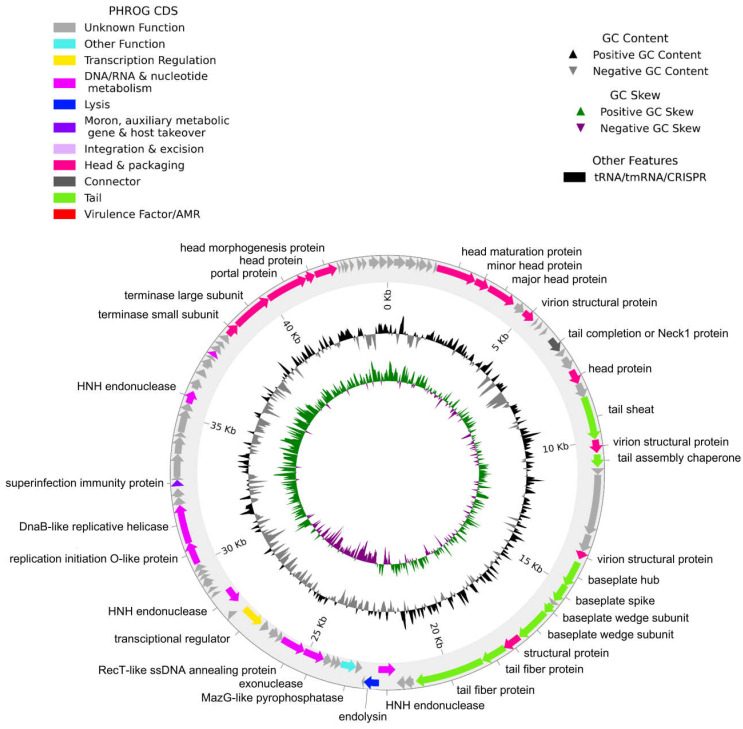
Circular genome map of Acinetobacter phage vB_AbaM_A72. Predicted ORFs are color-coded based on functional annotation according to the PHROG classification scheme. The outer ring displays annotated genes, with selected functional labels. The inner rings represent GC content (black) and GC skew (green for positive, purple for negative). Arrowheads indicate the direction of transcription.

Of the 92 coding sequences identified, only 33 were functionally annotated, while the remaining sequences were classified as hypothetical proteins with unknown function (Figure 4). The annotated genes were classified into eight categories: (1) proteins involved in capsid and packaging, (2) connector proteins, (3) tail proteins, (4) lysis proteins, (5) proteins involved in DNA/RNA and nucleotide metabolism, (6) transcription regulatory proteins, (7) moron proteins, auxiliary metabolic genes and host takeover, and (8) other functions (Table S2).

Notably, the genome of phage A72 harbors an endolysin gene that shares 96.47% identity with a glycoside hydrolase family 108 protein previously identified in *Escherichia coli* (WP_236516118.1), a key component in bacterial cell wall degradation and lysis.

**Figure 5:**
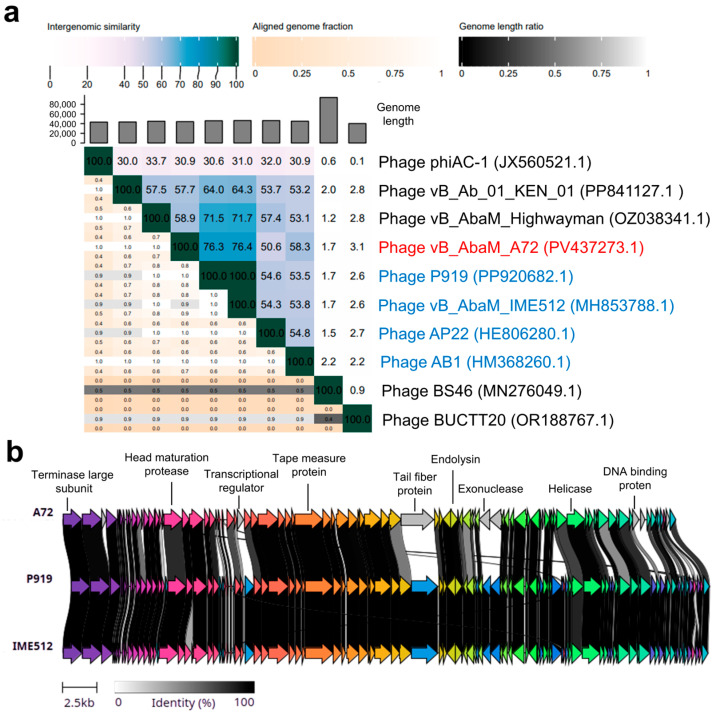
Comparative genomics of Acinetobacter phage vB_AbaM_A72 and its closest relatives. (**a**) Heatmap of intergenomic similarities between phage A72 and related phages, calculated using VIRIDIC. Phage names in blue correspond to members currently classified within the *Obolenskvirus* genus, while names in black represent unclassified members of the *Caudoviricetes* class. (**b**) Synteny plot comparing the genomes of phages A72, P919, and IME512, showing regions of nucleotide identity. Key genes and those with notable sequence discrepancies are labeled. Homologous genes are indicated by matching colors across genomes.

Figure 5a shows the intergenomic similarities in the form of a heatmap. Intergenomic similarities were calculated between different genomes available in blastn: phiAC-1, KEN_01, Highwayman, P919, IME512, AP22, AB1, BS46 and BUCTT20. Phages Acinetobacter phage P919 and Acinetobacter phage vB_AbaM_IME512 had the highest intergenomic similarity with phage A72, with 76.3% and 76.4%, respectively. It is worth noting that phages P919 and IME512 have 100% similarity, so they are considered the same bacteriophage. Likewise, more distant relationships were also observed with phages that infect *A. baumannii*. Based on these results, A72 is classified as a newly discovered phage of a new species within the *Obolenskvirus* genus, belonging to the *Caudoviricetes* class. The synteny plot revealed key genetic differences between A72 and its closest relatives, phages P919 and IME512 (Figure 5b). These differences were primarily found in genes encoding transcriptional regulators, exonucleases, DNA-binding proteins, and, most notably, a highly divergent tail fiber protein. This last finding is particularly significant, given the critical role of tail fibers in host receptor recognition. However, additional genomic information about the bacterial hosts susceptible to these phages is necessary to determine the functional implications of these structural variations.

**Figure 6:**
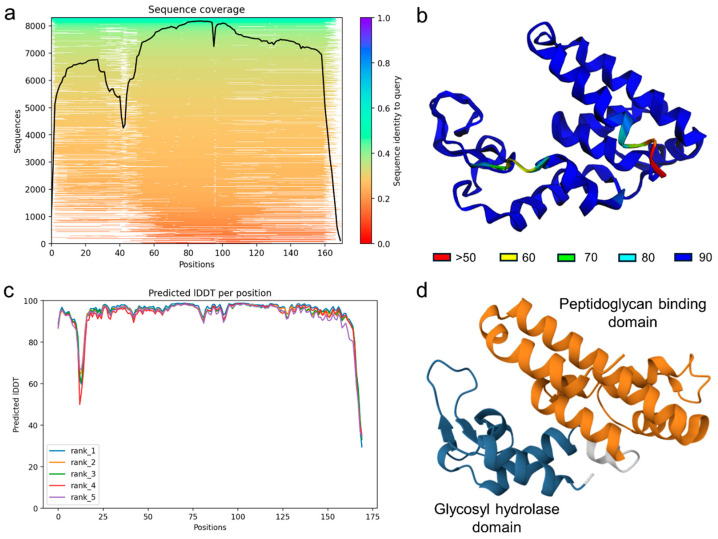
Bioinformatic characterization of an endolysin belonging to the glycosyl hydrolase family 108. (**a**) Multiple sequence alignment depth and sequence identity across the protein length, indicating coverage and conservation used for structure prediction. (**b**) Predicted 3D structure of the endolysin with color-coded confidence scores based on pLDDT. (**c**) Confidence scores per residue for the top five ranked models. (**d**) TED consensus domains of the glycosyl hydrolase 108 family proteins.

On the other hand, the predicted structure of the putative endolysin encoded by phage A72 exhibited high confidence and conservation across most of the protein sequence, as evidenced by robust multiple sequence alignment coverage and consistent confidence scores across structural models (Figure 6a,c). The predicted 3D model revealed two well-defined domains characteristic of glycosyl hydrolase family 108 proteins: a glycosyl hydrolase domain and a peptidoglycan-binding domain (Figure 6b,d). The glycosyl hydrolase domain is typically responsible for enzymatically cleaving bonds within the bacterial cell wall, facilitating host cell lysis during the phage replication cycle. The peptidoglycan-binding domain likely mediates substrate recognition and anchoring to the bacterial cell wall, enabling precise enzymatic action. The presence of both domains supports the classification of this protein as a functional endolysin and underscores its potential role in the lytic activity of the phage.

## 3. Discussion

Phages are the most abundant entities on Earth and are widely found in various environments, particularly in aquatic ecosystems [27]. In this work, phage A72 was isolated from stagnant water. The plaques formed by phages can vary in size, ranging from 1 to 7 mm, and may or may not exhibit a halo around the clear zone [28]. Recent studies have identified that the formation of halos around phages targeting *A. baumannii* is due to the presence of depolymerase enzymes. These enzymes can degrade the bacterial capsule, facilitating the access of the phage to its host cell [29]. The formation of these halos is considered a key indicator of tail spike protein activity and serves as a quick method for identifying phages that target bacterial capsules. Additionally, the size of these halos is influenced by the incubation time, as longer durations allow for greater diffusion and activity of depolymerases in the medium [30].

Phage therapy has re-emerged as a highly promising alternative to combat infections caused by MDR bacteria. In this context, the host range is a crucial parameter for determining whether a phage is a suitable candidate for phage therapy [24,31]. Ideally, a phage should exhibit a broad host range within the same bacterial species, thereby avoiding infection of other genera and helping to preserve the integrity of the patient’s microbiome. However, this feature can be counterproductive in certain cases, as bacteria may develop resistance to the phage, compromising therapeutic efficacy and necessitating alternative strategies such as the use of phage cocktails targeting different bacterial receptors [16,24].

Regarding the stability of phage A72 to environmental stress, our findings are consistent with those of Bagińska et al. [32], who examined the stability of 12 phages specific to *A. baumannii*. According to their research, a pH of 3 significantly inactivates virus particles, while a pH of 7 to 9 is ideal for preserving phage viability. This effect is attributed to the fact that bacteriophages are primarily composed of proteins, which denature under low pH conditions. On the other hand, our results regarding temperature align with previous research, such as Huang et al. [33], which reported that phage Abp95, a phage infecting *A. baumannii*, remained stable up to 60 °C and exhibited a comparable 100-fold reduction at 70 °C, mirroring the pattern observed in our study. External environmental factors play a crucial role in the viability and effectiveness of phage application, among them, temperature and pH [34]. However, in clinical applications, temperature variations within the human body are minimal, whereas pH can vary widely, with organs such as the stomach reaching values as low as 1.6. Therefore, the route of administration and the delivery system must be carefully considered to ensure that phages reach their target site while remaining viable [35].

Relating to the one-step growth curve, our results are consistent with Tan et al. [36], where phage vB_AbaM_AB3P2 showed a latent period of just 10 min, similar to the phage isolated in our study. In the same way, Jian et al. [37] conducted a study where Acinetobacter phage Abp9, which was isolated from sewage, showed a burst size of 158 viral particles per infected cell, such as A72. In terms of phage therapy, it is desirable for a phage to display desirable biological characteristics such as short latency periods and large burst sizes, as these properties allow the virus to spread more rapidly, thus reducing the proliferation of the bacteria [16].

The bacteriolytic activity of phages is important, and that is why other authors such as Wintachai & Voravuthikunchai [38] characterized a lytic bacteriophage belonging to the abolished *Myoviridae* family against MDR *A. baumannii* strains. These authors report that the phage was able to control the growth of the host strain for up to 10 h at an MOI of 1. However, when assessed at 24 h, they observed an increase in optical density. In our study, we evaluated the bacteriolytic activity of phage A72 over a 40 h period and observed sustained lysis, particularly at an MOI of 100. Notably, even lower MOIs demonstrated a satisfactory suppression index, indicating effective bacterial control across a range of phage concentrations. This prolonged activity is especially relevant given that other phages have been reported to permit the emergence of phage resistance within 4 to 7 h post-infection [23,39]. These results underscore the therapeutic potential of phage A72, as its sustained lytic effect may help delay or reduce the likelihood of resistance development during treatment.

Other authors have reported phages with similar genomic characteristics as A72 [40]. Such is the case for phage BUCT629, which was isolated from hospital sewage and was shown to have a linear double-stranded DNA sequence with a length of 46,325 bp and a G+C content of 38%, similar to phage A72. For a phage to be considered safe for administration, some organizations, such as the ARLG Phage Taskforce, have suggested that phages used in phage therapy should not contain antibiotic resistance genes, virulence genes, toxin genes, or genes related to lysogeny (e.g., integrase genes, integrase regulatory genes, among others) [41]. This underscores the critical role of whole-genome sequencing in the comprehensive characterization of therapeutic phages. It is important to note that, as with many phages, a substantial portion of A72′s ORFs remain annotated as hypothetical proteins, reflecting current limitations in genome annotation pipelines and highlighting the potential for uncharacterized genes to contribute to phage biology and host interactions.

Finally, our phage encodes a putative endolysin with a conserved domain architecture typical of glycosyl hydrolase family 108, similar to endolysins previously reported in *Obolenskvirus* phages [42]. Structural modeling revealed an N-terminal glycosyl hydrolase domain, which likely acts as a lysozyme responsible for cleaving bonds in the peptidoglycan layer. This domain includes a conserved EGGY motif, with a glutamic acid residue essential for catalytic activity [43]. At the C-terminus, a putative peptidoglycan-binding domain was identified, which may facilitate substrate recognition and anchoring to the bacterial cell wall, enhancing the enzymatic action of the catalytic domain [44]. These findings suggest that the endolysin contributes to bacterial cell wall degradation during the final stage of the phage lytic cycle, enabling efficient progeny release [45,46]. Endolysins with similar dual-domain architecture have been explored as antimicrobial agents, either alone or in engineered combinations targeting multiple cleavage sites to enhance synergy and reduce the required dose [47].

## 4. Materials and Methods

### 4.1. Biological Material

The biological material used in this study was provided by the Hospital Civil de Guadalajara Fray Antonio Alcalde. The strains were identified using Vitek 2, as was their antibiotic resistance profile (Table S1).

### 4.2. Phage Isolation and Transmission Electron Microscopy

For phage isolation, water samples were taken from stagnant water in Jalisco, Mexico (20°32′31.81″ N, 102°47′31.99″ W), using the protocol described by González-Gómez et al. [48] with few modifications. Briefly, 200 mL of samples were incubated overnight at 37 °C in 200 mL of Trypticase Soy Broth 2X (TSB) without any prior treatment and 1 mL of *A. baumannii* strain A72 that was isolated from an endotracheal aspirate. After incubation overnight, samples were centrifuged (10,000× *g*, 10 min and 4 °C) and the supernatant was filtered twice using PES syringe filters with pore sizes of 0.45 µm and 0.22 µm (Cytiva Whatman, MA, USA), respectively. The filtrate obtained was used to detect the presence of lytic phages infecting this MDR *A. baumannii* strain, through a spot test. For phage purification, the double-layer agar technique was used with serial dilutions of the phage, which were diluted with SM buffer (100 mM NaCl, 8 mM MgSO_4_, and 50 mM Tris-HCl, pH 7.5). Briefly, 100 µL of the suspension was added to 3 mL of soft agar and poured onto a previously solidified TSA plate. A well-isolated plaque was selected, and the process was repeated three times.

The purified phage was propagated according to the protocol described by Jamalluden et al. [49]. Briefly, five Petri dishes were incubated overnight at 37 °C using the double-layer agar method previously described. Next day, 5 mL of SM buffer was added to each plate and gently agitated for 15 min to dissolve the soft agar layer. The resulting suspension was collected into a sterile conical tube, centrifuged, and filtered. The phage titer was then determined using the double-layer agar technique.

The isolated phage was analyzed by transmission electron microscopy (TEM) using González-Gómez et al. [50] protocol with a few modifications. Briefly, a drop of the phage suspension was mounted on a 300-mesh copper grid and stained with 2% uranyl acetate (Electron Microscopy Science, Hatfield, PA, USA) dissolved in deionized water. Micrographs were taken at various magnifications using a Morgagni M-268 transmission electron microscope (Philips/FEI, Amsterdam, The Netherlands). We select five phages’ micrographs to determine the capsid and tail dimensions expressed as the mean ± standard deviation.

### 4.3. Host Range and Efficiency of Plating

The host range of the phage was determined by spot test following the protocol by Asghar et al. [51], with a few modifications. This evaluation was carried out against nineteen MDR clinical strains of *A. baumannii* (additional data are given in Table S1) and other bacterial genera *S. aureus* ATCC 6538, *S. aureus* ATCC 25923, *E. coli* ATCC 11303, *Salmonella enterica* ATCC 10708, *P. aeruginosa* ATCC 15,442 and *L. monocytogenes* ATCC 19,111 (Table 1) in order to assess the phage’s host range across different bacterial genera. Briefly, 100 µL of the host bacteria were mixed with 3 mL of soft agar, poured onto a previously prepared TSA dish, and allowed to solidify. Once solidified, 10 µL of the undiluted phage suspension were applied on the surface of the agar, allowing them to dry at room temperature, and then incubated overnight at 37 °C. The following day, plates were examined for zones of lysis, confirming the infectivity of the phage against the tested strain. In addition, strains were further confirmed for phage infectivity by calculating the efficiency of plating (EOP) spotting different dilutions. EOP was calculated as the ratio between the determined PFU on the test strain and the original host strain, and the strains were interpreted for phage infectivity based on EOP values as high (++, EOP ≥ 0.5), moderate (+, EOP between 0.1 and 0.5) and low (−, EOP ≤ 0.1).

### 4.4. Stability Assays: Temperature and pH

Stability assays were conducted by subjecting the phage to different temperature and pH ranges, following the Capra et al. [52] protocol. Briefly, 100 µL of the phage stock was incubated for 1 h in a dry bath (Labnet International, Inc., Edison, NJ, USA) at various temperatures (30, 37, 40, 50, 60, 70 and 80 °C). Similarly, 100 µL of the phage stock was mixed with 900 µL of SM buffer at different pH values (3, 5, 7.5, 9 and 11) and incubated at 37 °C for 1 h. After pH and thermal treatment, the determination of the phage titers was conducted using the double-layer agar technique.

### 4.5. One-Step Growth Curve

The one-step growth curve assay was conducted based on the protocol by Kropinski [53] with few modifications. In brief, a phage-bacteria mixture was prepared at a multiplicity of infection (MOI) of 0.1. The host strain culture was adjusted to 6 log_10_ CFU/mL in 10 mL of TSB and mixed with the phage stock to obtain a final concentration of 10^5^ PFU/mL. Following a 15 min adsorption period at 37 °C, the mixture was centrifuged at 12,000× *g* for 2 min. The supernatant, containing unabsorbed phages, was discarded, and the pellet was resuspended in 10 mL of TSB. At time point 0, two samples were collected, with chloroform added to one of them. The mixture was then incubated at 37 °C, with samples taken every 10 min for a total of 70 min and analyzed using the soft agar overlay method. The burst size was calculated by dividing the final titer of released viral particles by the initial titer of infected cells.

### 4.6. Bacteriolytic Activity

This experiment was performed using a 96-well microplate system with *A. baumannii* as the host, as described by González-Gómez et al. [48]. An overnight bacterial culture was adjusted to an OD of 0.11 (~6 log_10_ CFU/mL) and used as the inoculum. Phage dilutions were prepared to achieve MOIs of 100, 10, 1, 0.1 and 0.01. Each well was initially filled with 100 µL of TSB. Experimental wells received an additional 80 µL of the corresponding phage dilution to achieve the desired multiplicity of infection (MOI), followed by 20 µL of bacterial culture, resulting in a final volume of 200 µL per well. Negative controls (TSB only) and positive controls (TSB with bacterial strain) were included to validate the assay. The microplate was incubated at 37 °C for 40 h in a microplate reader (Thermo Scientific, Waltham, MA, USA) with absorbance measurements at 600 nm taken every 30 min, including shaking before each reading. The experiment was performed in triplicate. At the end of the incubation period, the area under the curve (AUC) was calculated for each MOI and for the positive control. The suppression index was then determined using the formula (AUC of positive control—AUC of MOI)/AUC of positive control, which quantifies the degree of bacterial growth suppression induced by the phage treatment [54].

### 4.7. DNA Extraction and Genome Sequencing

Phage DNA was extracted using the phenol-chloroform method as described by Pickard [55] with few modifications. Briefly, 900 µL of phage suspension was transferred to a 1.5 mL microtube, and 9 µL of DNase (1 mg/mL) and 4 µL of RNase A (10 mg/mL) were added. The mixture was vortexed and incubated at 37 °C for 30 min. Following this, 46 µL of SDS (10%) and 9 µL of proteinase K (10 mg/mL) were added, and the mixture was incubated at 37 °C for 30 min, followed by incubation at 56 °C for 30 min. An equal volume of phenol/chloroform/isoamyl alcohol (25:24:1) was added to the sample, mixed thoroughly, and centrifuged at 8000× *g* for 10 min. The aqueous phase was carefully transferred to new microtubes, and an equal volume of chloroform/isoamyl alcohol (24:1) was added. The tubes were centrifuged again at 8000× *g* for 10 min, and the aqueous phase was collected and combined with 45 µL of 3M sodium acetate and 500 µL of isopropanol. The samples were incubated overnight at −20 °C, followed by centrifugation at 20,000× *g* for 10 min. The supernatant was discarded, and the DNA pellet was washed with ice-cold 70% ethanol. Finally, the pellet was air-dried, resuspended in TE buffer, and stored at −20 °C.

Genomic libraries were prepared using the Nextera XT Library Preparation Kit (Illumina, San Diego, CA, USA) following the manufacturer’s recommendations. Libraries were quantified using a Qubit 2.0 fluorometer (Thermo Fischer Scientific, Waltham, MA, USA), and genomic sequencing was performed on the Illumina MiniSeq platform using a 2 × 150 bp paired-end protocol for 300 cycles. A total of 116,462 raw reads with a combined length of 12,144,508 bp were generated and subsequently quality-filtered using fastp v0.23 [56,57] and assembly was carried out using SPAdes v3.13.0 [58], with a 10× coverage threshold to retain contigs. The final assembly achieved 81× coverage and was validated by mapping reads against reference assemblies using Geneious v9.1.8.

### 4.8. Bioinformatic Analyses

The structural and functional annotation was performed using Pharokka v1.7.5 [59]. The detection of transfer RNA (tRNA) was conducted using the ARAGORN search engine [60]. Virulence genes were searched using the VFDB database [61], while antibiotic resistance genes were searched using CARD [62] and AMRFinder Plus v4.0.19 [63]. Phage life cycle classification was performed using Phage AI v1.0.2, an artificial intelligence-based software platform developed by Tynecki et al. [64]. Intergenomic similarities between different phage genomes were calculated using VIRIDIC [65] with default settings in blastn. A synteny plot was generated using clinker v0.0.31 [66], comparing the genome of phage vB_AbaM_A72 with the two most homologous phage genomes, as identified by the VIRIDIC intergenomic similarity analysis.

The putative endolysin encoded in the genome of phage vB_AbaM_A72 was structurally modeled using ColabFold v1.5.5 [67], which implements AlphaFold2 for high-accuracy protein structure prediction. Domain architecture was subsequently identified using InterPro v106.0 [68], incorporating annotations from The Encyclopedia of Domains (TED) database [69], to classify conserved functional regions.

### 4.9. Statistical Analysis

The experiments were performed in triplicate, and the results are shown as the mean value with the standard deviation. Statistical analysis was conducted using STATGRAPHICS Centurion XVI v16.1.03. To determine significant differences, a one-way analysis of variance (ANOVA) followed by Fisher’s Least Significant Difference test was used. Statistical significance was set at *p* < 0.05.

## 5. Conclusions

In conclusion, this phage represents a newly described species within the genus *Obolenskvirus*, as only two previously reported viruses share greater than 70% genomic similarity. The phenotypic and genomic characterization of the Acinetobacter phage vB_AbaM_A72 has demonstrated that it meets the desirable features for a phage to be used in phage therapy. This is of utmost importance in phage therapy, as large libraries of characterized phages with diverse host ranges are required to cover the vast world of strain varieties.

## Figures and Tables

**Table 1:** Host range and efficiency of plating (EOP) of Acinetobacter phage vB_AbaM_A72 against different strains.

Strain	Code	Source	Spot Assay	EOP
*A. baumannii*	A089	Peritoneal fluid	-	-
*A. baumannii*	A103	Urine	-	-
*A. baumannii*	A048	Lung aspirate	-	-
*A. baumannii*	A045	Endotracheal aspirate	-	-
*A. baumannii*	A063	Surgical wound	-	-
*A. baumannii*	A137	Sputum	-	-
*A. baumannii*	A052	Endotracheal aspirate	-	-
*A. baumannii*	A062	Urine	-	-
*A. baumannii*	A038	Sputum	-	-
*A. baumannii*	A007	Blood culture	-	-
*A. baumannii*	A064	Urine	-	-
*A. baumannii*	A095	Blood culture	-	-
*A. baumannii*	A071	Endotracheal aspirate	-	-
*A. baumannii*	A098	Endotracheal aspirate	-	-
*A. baumannii*	A056	Blood culture	-	-
*A. baumannii*	A067	Endotracheal aspirate	-	-
*A. baumannii*	A075	Endotracheal aspirate	+	++ (1.00)
*A. baumannii*	A019	Urine	+	++ (0.78)
*A. baumannii*	A069	Urine	-	-
*S. aureus*	ATCC 6538	Human lesion	-	-
*S. aureus*	ATCC 25923	Clinical isolate	-	-
*E. coli*	ATCC 11303	Not described	-	-
*S. enterica*	ATCC 10708	Not described	-	-
*P. aeruginosa*	ATCC 15442	Water bottle	-	-
*L. monocytogenes*	ATCC 19111	Poultry	-	-

++: complete lysis; +: partial lysis; -: no lysis.

## Data Availability

The Acinetobacter phage vB_AbaM_A72 genome reported here is available in the GenBank database under the accession number PV437273.1.

## Supplementary Materials

The following supporting information can be downloaded at https://www.mdpi.com/article/10.3390/antibiotics14100961/s1: Figure S1: Morphology of phage A72 seen under Transmission Electronic Microscopy (TEM) stained with 2% uranyl acetate; Table S1: Antibiotic resistance profile of *A. baumannii* clinical isolates according to Vitek 2; Table S2: Genomic features of Acinetobacter phage vB_AbaM_A72.
