# Supplementary material for: A Newly Discovered Obolenskvirus Phage with Sustained Lytic Activity Against Multidrug-Resistant Acinetobacter baumannii
Source: Antibiotics (Basel). 2025 Sep 24;14(10):961. doi: 10.3390/antibiotics14100961 (PMC12561047; doi:10.3390/antibiotics14100961)
Supplement: Supplementary file 1 [file antibiotics-14-00961-s001.zip › Supplementary Material 2.pdf]

Figure S1. Morphology of phage A72 seen under Transmission Electronic Microscopy (TEM) stained with 2% uranyl acetate.

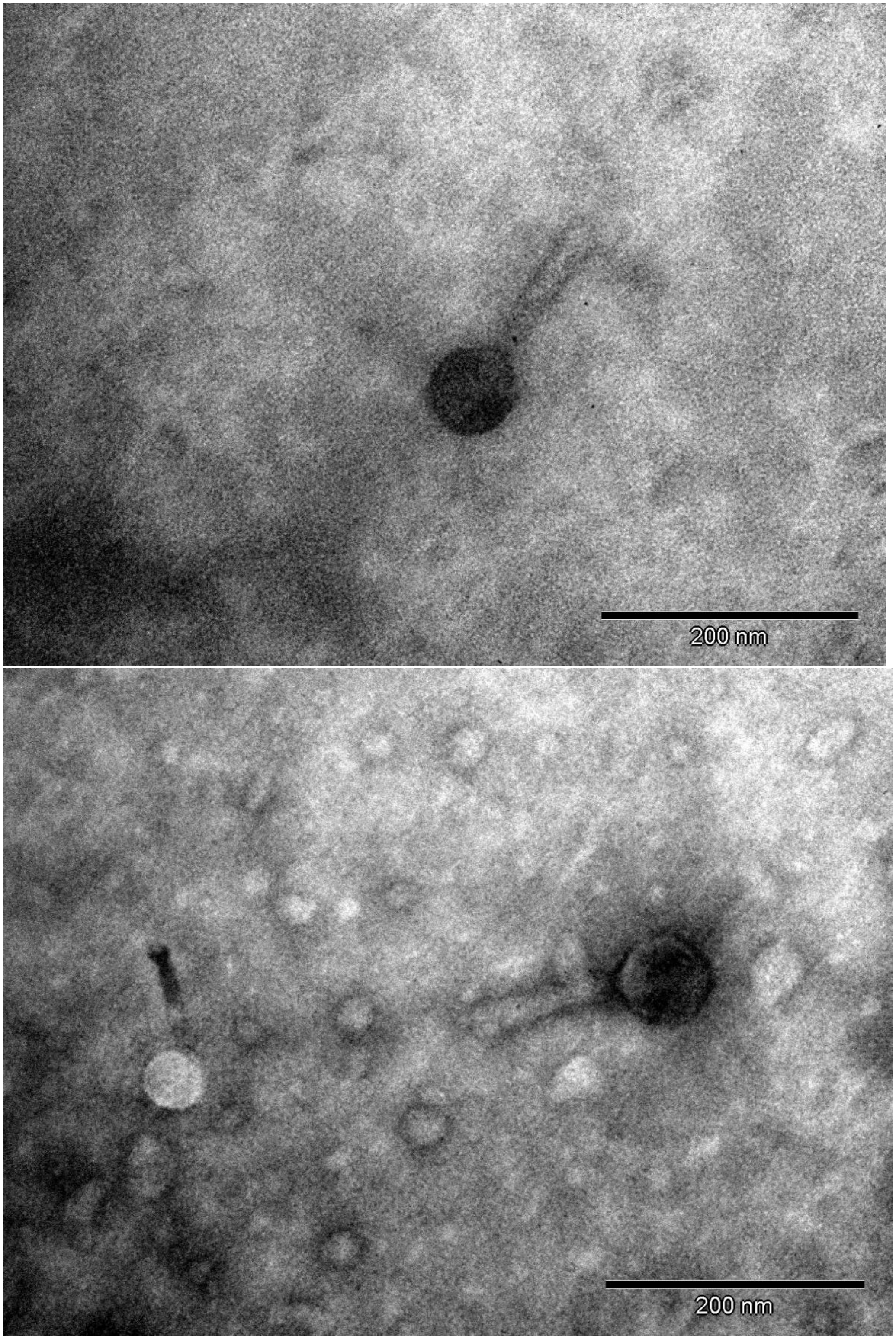

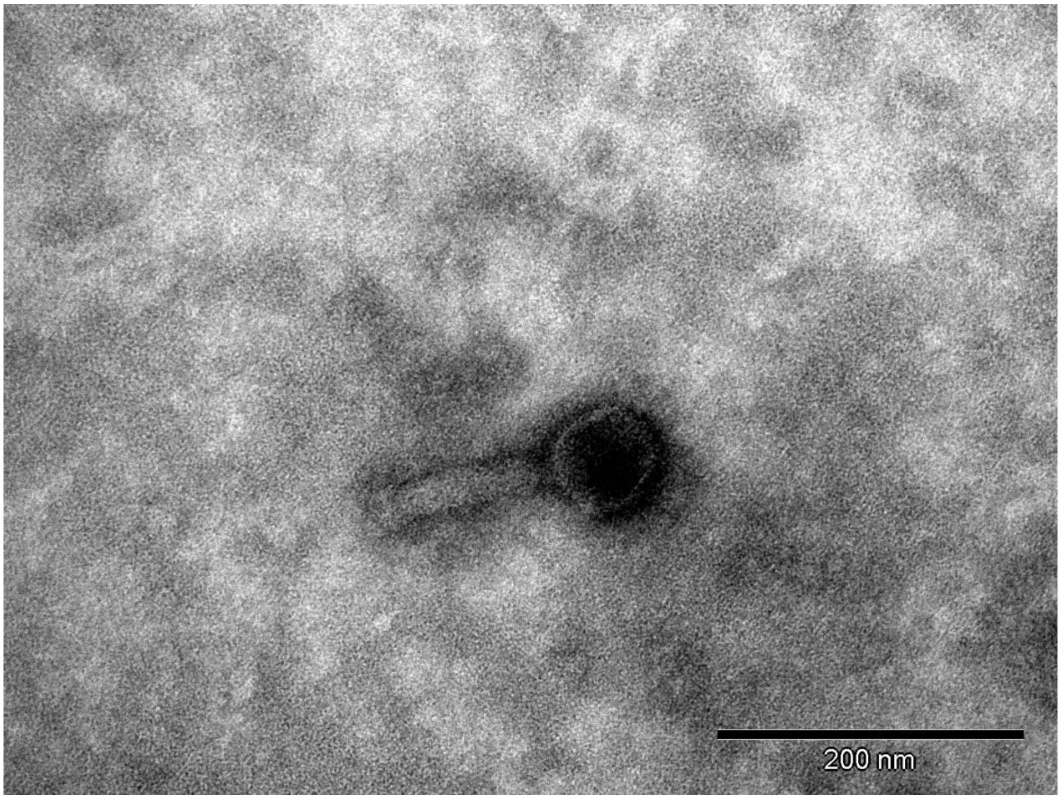
200 nm

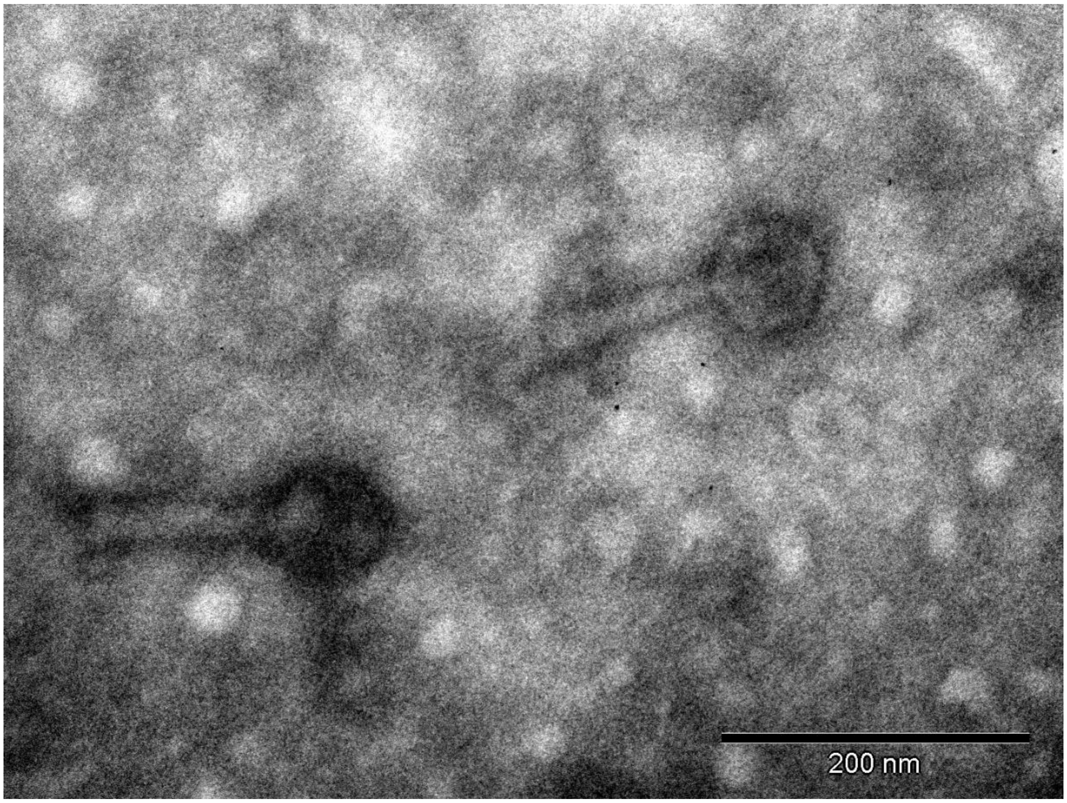
200 nm
