# Supplementary material for: A Newly Discovered Obolenskvirus Phage with Sustained Lytic Activity Against Multidrug-Resistant Acinetobacter baumannii
Source: Antibiotics (Basel). 2025 Sep 24;14(10):961. doi: 10.3390/antibiotics14100961 (PMC12561047; doi:10.3390/antibiotics14100961)
Supplement: Supplementary file 1 [file antibiotics-14-00961-s001.zip › Supplementary tables 1 and 2.pdf]

Table S1. Antibiotic resistance profile of *A. baumannii* clinical isolates according to Vitek 2.

Strain	Source	Antibiotic										
		SAM	CAZ	CRO	CIP	GEN	IPM	TZP	FEP	MEM	TIG	DOR
A089	Peritoneal fluid	R	R	R	R	R	R	R	R	R	I	R
A103	Urine	R	R	R	R	R	R	R	R	R	I	R
A048	Lung aspirate	R	R	R	R	I	R	R	R	R	S	R
A045	Endotracheal aspirate	R	R	R	R	I	R	R	R	R	S	R
A063	Surgical wound	R	R	R	R	R	R	R	R	R	S	R
A137	Sputum	R	R	R	R	R	R	R	R	R	I	R
A052	Endotracheal aspirate	R	R	R	R	R	R	R	R	R	S	R
A062	Urine	R	R	R	R	R	R	R	R	R	S	R
A038	Sputum	I	R	R	R	S	R	R	R	R	S	R
A007	Blood culture	I	R	R	R	S	R	R	R	R	I	R
A064	Urine	R	R	R	R	S	R	R	R	R	S	R
A095	Blood culture	I	R	R	R	S	R	R	R	R	S	R
A071	Endotracheal aspirate	R	R	R	R	S	R	R	R	R	S	R
A098	Endotracheal aspirate	I	R	R	R	S	R	R	R	R	S	R
A056	Blood culture	I	R	R	R	S	R	R	R	R	S	R
A067	Endotracheal aspirate	R	R	R	R	R	R	R	R	R	S	R
A075	Endotracheal aspirate	R	R	R	R	R	R	R	R	R	I	R
A019	Urine	R	R	R	R	R	R	R	R	R	I	R
A069	Urine	R	R	R	R	S	ND	ND	R	R	ND	ND
A72	Endotracheal aspirate	R	R	R	R	R	R	R	R	R	I	R

R: Resistant; I: Intermediate; S: Susceptible; ND: Not described; SAM: Ampicillin/Sulbactam; CAZ: Ceftazidime; CRO: Ceftriaxone; CIP: Ciprofloxacin; GEN: Gentamicin; IPM: Imipenem; TZP: Piperacillin/Tazobactam; FEP: Cefepime; MEM: Meropenem; TIG: Tigecycline; DOR: Doripenem

Table S2. Genomic features of Acinetobacter phage vB_AbaM_A72

Name	Minimum	Maximum	Length	Direction	Product
hypothetical protein CDS	223	615	393	forward	hypothetical protein
hypothetical protein CDS	612	974	363	forward	hypothetical protein
hypothetical protein CDS	978	1,076	99	forward	hypothetical protein
hypothetical protein CDS	1,073	1,348	276	forward	hypothetical protein
hypothetical protein CDS	1,338	1,523	186	forward	hypothetical protein
hypothetical protein CDS	1,582	1,692	111	forward	hypothetical protein
head maturation protease CDS	1,679	3,031	1,353	forward	head maturation protease
minor head protein CDS	3,031	3,525	495	forward	minor head protein
major head protein CDS	3,536	4,528	993	forward	major head protein
hypothetical protein CDS	4,608	4,958	351	forward	hypothetical protein
virion structural protein CDS	4,961	5,383	423	forward	virion structural protein
hypothetical protein CDS	5,435	5,524	90	forward	hypothetical protein
hypothetical protein CDS	5,611	5,733	123	forward	hypothetical protein
hypothetical protein CDS	5,861	5,974	114	forward	hypothetical protein
tail completion or Neck1 protein CDS	6,226	6,732	507	forward	tail completion or Neck1 protein
hypothetical protein CDS	6,747	6,935	189	forward	hypothetical protein
hypothetical protein CDS	7,001	7,435	435	forward	hypothetical protein
head protein CDS	7,483	7,986	504	forward	head protein
hypothetical protein CDS	7,983	8,477	495	forward	hypothetical protein
tail sheath CDS	8,467	9,930	1,464	forward	tail sheath
virion structural protein CDS	9,943	10,392	450	forward	virion structural protein
tail assembly chaperone CDS	10,438	10,863	426	forward	tail assembly chaperone
hypothetical protein CDS	10,893	11,105	213	forward	hypothetical protein
hypothetical protein CDS	11,108	13,138	2,031	forward	hypothetical protein
hypothetical protein CDS	13,146	13,742	597	forward	hypothetical protein
virion structural protein CDS	13,744	14,022	279	forward	virion structural protein

baseplate hub CDS	14,131	15,021	891	forward	baseplate hub
baseplate spike CDS	15,002	15,649	648	forward	baseplate spike
hypothetical protein CDS	15,652	15,798	147	forward	hypothetical protein
baseplate wedge subunit CDS	15,795	16,148	354	forward	baseplate wedge subunit
baseplate wedge subunit CDS	16,145	17,329	1,185	forward	baseplate wedge subunit
structural protein CDS	17,329	17,955	627	forward	structural protein
tail fiber protein CDS	17,933	18,802	870	forward	tail fiber protein
tail fiber protein CDS	18,804	21,134	2,331	forward	tail fiber protein
hypothetical protein CDS	21,210	21,530	321	forward	hypothetical protein
hypothetical protein CDS	21,514	21,777	264	forward	hypothetical protein
HNH endonuclease CDS	21,797	22,408	612	reverse	HNH endonuclease
endolysin CDS	22,370	22,882	513	forward	endolysin
hypothetical protein CDS	22,866	22,955	90	forward	hypothetical protein
hypothetical protein CDS	22,981	23,211	231	reverse	hypothetical protein
MazG-like pyrophosphatase CDS	23,204	23,761	558	reverse	MazG-like pyrophosphatase
hypothetical protein CDS	23,761	23,952	192	reverse	hypothetical protein
hypothetical protein CDS	23,922	24,104	183	reverse	hypothetical protein
hypothetical protein CDS	24,101	24,394	294	reverse	hypothetical protein
exonuclease CDS	24,395	25,150	756	reverse	exonuclease
RecT-like ssDNA annealing protein CDS	25,147	26,046	900	reverse	RecT-like ssDNA annealing protein
hypothetical protein CDS	26,043	26,225	183	reverse	hypothetical protein
hypothetical protein CDS	26,225	26,557	333	reverse	hypothetical protein
hypothetical protein CDS	26,650	26,907	258	reverse	hypothetical protein
transcriptional regulator CDS	26,974	27,786	813	reverse	transcriptional regulator
hypothetical protein CDS	27,886	28,080	195	forward	hypothetical protein
HNH endonuclease CDS	28,133	28,720	588	reverse	HNH endonuclease
hypothetical protein CDS	28,775	28,894	120	forward	hypothetical protein
hypothetical protein CDS	28,963	29,103	141	forward	hypothetical protein
hypothetical protein CDS	29,153	29,476	324	forward	hypothetical protein

hypothetical protein CDS	29,461	29,586	126	forward	hypothetical protein
hypothetical protein CDS	29,599	29,778	180	forward	hypothetical protein
hypothetical protein CDS	29,775	29,987	213	forward	hypothetical protein
replication initiation O-like CDS	29,997	30,752	756	forward	replication initiation O-like
DnaB-like replicative helicase CDS	30,737	32,053	1,317	forward	DnaB-like replicative helicase
hypothetical protein CDS	32,054	32,320	267	forward	hypothetical protein
hypothetical protein CDS	32,317	32,607	291	forward	hypothetical protein
immunity to superinfection CDS	32,680	32,892	213	forward	immunity to superinfection
hypothetical protein CDS	32,889	33,002	114	forward	hypothetical protein
hypothetical protein CDS	32,990	33,757	768	forward	hypothetical protein
hypothetical protein CDS	33,754	34,329	576	forward	hypothetical protein
hypothetical protein CDS	34,326	34,490	165	forward	hypothetical protein
hypothetical protein CDS	34,487	35,251	765	forward	hypothetical protein
hypothetical protein CDS	35,238	35,504	267	forward	hypothetical protein
HNH endonuclease CDS	35,485	35,931	447	forward	HNH endonuclease
hypothetical protein CDS	36,014	36,364	351	forward	hypothetical protein
hypothetical protein CDS	36,502	36,741	240	forward	hypothetical protein
hypothetical protein CDS	36,805	37,152	348	forward	hypothetical protein
hypothetical protein CDS	37,149	37,271	123	forward	hypothetical protein
HTH DNA binding protein CDS	37,264	37,455	192	forward	HTH DNA binding protein
hypothetical protein CDS	37,452	37,694	243	forward	hypothetical protein
hypothetical protein CDS	37,684	37,848	165	forward	hypothetical protein
hypothetical protein CDS	37,905	38,141	237	forward	hypothetical protein
terminase small subunit CDS	38,145	38,585	441	forward	terminase small subunit
terminase large subunit CDS	38,585	39,958	1,374	forward	terminase large subunit
portal protein CDS	39,961	41,388	1,428	forward	portal protein
head protein CDS	41,381	41,704	324	forward	head protein
head morphogenesis CDS	41,707	42,477	771	forward	head morphogenesis
hypothetical protein CDS	42,506	42,598	93	forward	hypothetical protein

hypothetical protein CDS	42,629	42,718	90	forward	hypothetical protein
hypothetical protein CDS	42,719	42,874	156	forward	hypothetical protein
hypothetical protein CDS	42,915	43,049	135	forward	hypothetical protein
hypothetical protein CDS	43,167	43,331	165	forward	hypothetical protein
hypothetical protein CDS	43,332	43,478	147	forward	hypothetical protein
hypothetical protein CDS	43,560	43,913	354	forward	hypothetical protein
